# Reactive leukocytosis in older patients with acute colonic diverticulitis: A retrospective study utilizing logistic regression analysis

**DOI:** 10.1111/ggi.14027

**Published:** 2020-09-02

**Authors:** Yosuke Sasaki, Fumiya Komatsu, Naoyasu Kashima, Tadashi Maeda, Yoshihisa Urita

**Affiliations:** ^1^ Department of General Medicine and Emergency Care, School of Medicine Toho University Tokyo Japan

**Keywords:** diverticulitis, geriatrics, immunosenescence, leukocytosis, neutrophilia

## Abstract

**Aim:**

Although various neutrophilic immunosenescence mechanisms have been shown, there are few clinical studies on age‐related differences in leukocytosis against acute bacterial infections, including acute colonic diverticulitis.

**Methods:**

We performed a retrospective study of 26 patients ≥65 years old and 211 patients 16–64 years old who were hospitalized for acute colonic diverticulitis at Toho University Medical Center Omori Hospital between 2010 and 2016. We compared patients' characteristics, including sex, immunocompromised status, diverticulitis site, complications, severity, previous diverticulitis, vital signs, leukocyte counts, neutrophil‐to‐lymphocyte ratio and serum C‐reactive protein on admission. To adjust for confounding factors, we performed a logistic regression analysis.

**Results:**

Univariate comparisons showed that leukocyte count (older: 10 850 [interquartile range, 9400–12 000]/mm^3^ vs. younger: 12 600 [interquartile range, 10 500–15 000]/mm^3^, *P* = 0.004) and prevalence of leukocytosis (leukocytes >11 000/mm^3^) were lower in older compared with younger patients. There were significantly more female, left‐sided diverticulitis and immunocompromised patients in the older compared with the younger group. Logistic regression showed that leukocyte count, prevalence of female patients, and left‐sided diverticulitis were independent predictors for the older patients: Their odds ratios were 0.866 (95% confidence interval [95% CI] 0.753–0.996), 2.631 (95% CI, 1.032–6.707) and 5.810 (95% CI, 2.328–14.497), respectively.

**Conclusion:**

Caution should be taken when managing older patients with colonic diverticulitis because reactive leukocytosis might be poor, possibly reflecting immunosenescence. **Geriatr Gerontol Int 2020; 20: 951–955**.

## Introduction

Because of the recent progress in studies on immunosenescence, various mechanisms of neutrophilic immunosenescence have been reported, including reduced cytokine signaling, peroxide/nitric oxide production, phagocytic function,^1^, ^2^ shortened half‐life time because of altered Jak–STAT activation,^3^, ^4^ decreased T‐cell receptor ligand repertoire diversity,^5^ impaired neutrophil migration because of increased constitutive phosphoinositide 3‐kinase signaling^6^ and impaired neutrophil extracellular traps.^7^

However, there are few clinical studies on the age‐related difference of leukocytosis in response to bacterial infection. Previous studies on age‐related differences in reactive leukocytosis in patients with acute bacterial infections are heterogeneous and their results are inconsistent^8^, ^9^, ^10^; the inclusion criteria and definitions of older patients differed considerably. Thus, we would like to examine whether older patients have poorer reactive leukocytosis against acute colonic diverticulitis, as an example of acute bacterial infection. Therefore, we performed a retrospective study that utilized a combination of previously collected data from a retrospective study on clinical differentiation of appendicitis and acute colonic diverticulitis (ACD) of the right‐sided colon,^11^ and additionally corrected data for the present study.

## Methods

### *Design and patients*

In this retrospective study, we evaluated the medical records from patients who were hospitalized for ACD between January 2010 and December 2016 at Toho University Medical Center Omori Hospital. The hospital has 948 beds and is located in Tokyo, Japan. Three authors (YS, FK and KN) reviewed patients' records and enrolled patients who were appropriately diagnosed with ACD based on their clinical findings, laboratory test results and abdominal computed tomography scan findings. For patients who were hospitalized since 2012, we utilized the data that were initially collected for another previously published study on clinical differentiation of diverticulitis and appendicitis.^11^ For patients who were admitted between 2010 and 2011, we additionally collected data for the present study.

The center's ethics committee approved the study protocol (M17057) and waived the requirement for individual informed consent subject to public announcement of the research because of the retrospective and non‐invasive study design.

### *Study variables*

We collected data including age, sex, ACD side (right‐sided colon vs. left‐sided colon), complications of diverticulitis such as perforation and/or abscess, vital signs (e.g., blood pressure, heart rate and body temperature) and leukocyte counts on admission. We reviewed underlying immunocompromised status, including cancer, diabetes mellitus, liver cirrhosis, end‐stage renal disease and immunosuppressant use (including corticosteroids) of all participants because the immunocompromised status is generally thought to affect neutrophilic function and a previous study reported that immunosuppressant use was associated with normal leukocyte counts although corticosteroid use may induce leukocytosis.^12^ We also monitored the neutrophil‐to‐lymphocyte ratio if it was recorded. To evaluate the prevalence of leukocytosis between old and young groups, we defined leukocytosis as a leukocyte count >11 000/mm^3^, in accordance with a previous study.^13^ We also defined abnormal vital signs based on previous studies: fever was defined as a body temperature of ≥38.0°C,^14^ shock as systolic blood pressure <12.0 kPa (<90 mmHg),^15^ and tachycardia as a heart rate ≥100 beats/min. We defined complicated diverticulitis as a case in which abscess or perforation was detected by ultrasound or computed tomography scan. We subsequently categorized the severity of cases based on the classification proposed by Sallinen *et al*. as follows: 1, uncomplicated diverticulitis; 2, complicated diverticulitis with small abscesses (<6 cm); 3, complicated diverticulitis with large abscess (≥6 cm) or distant intraperitoneal or retroperitoneal gas; 4, generalized peritonitis without organ dysfunction; and 5, generalized diverticulitis with organ dysfunction.^16^ According to the previously reported predictors of severe diverticulitis, we also reviewed the use of non‐steroidal anti‐inflammatory drugs, past episodes of diverticulitis, and serum C‐reactive protein for adjustment of confounding due to severity.^17^ We also evaluated blood culture results if submitted.

### *Statistical analyses*

In accordance with the previous study, we divided patients into the following two groups: older patients (age ≥65 years old; *n* = 26) and younger patients (age 16–64 years old; *n* = 211).^18^ We performed statistical analyses to compare the characteristics of these two groups. All statistical analyses were performed using Stata/IC software (version 15.1; Stata Corp., College Station, TX, USA).

#### *Univariate comparisons*

We compared clinical characteristics between older and younger patients. The chi‐squared test was used for dichotomous/categorical variables, and the Wilcoxon rank‐sum test was utilized for continuous variables because the distribution distributions were skewed.

#### *Logistic regression analysis and evaluations of the model*

To adjust for confounding variables, we subsequently performed logistic regression analysis. We selected all variables that had a significant difference (*P* < 0.05) in the univariate comparisons as explanatory variables. We examined the variance inflation factors (VIFs) to evaluate the multicollinearity of the regression models. We also used a receiver operating characteristic analysis to evaluate the discrimination ability of the regression model. We calibrated the models using the Hosmer–Lemeshow goodness‐of‐fit test and performed internal validation using bootstrap methods involving 100 samples that were tested five times.

## Results

In total, 237 patients consisting of 26 (11.0%) older patients with a mean age of 75.5 years (interquartile range [IQR], 67.0–80.0) and 211 younger patients with a mean age of 41.0 years (IQR, 32.0–50.0) were evaluated. Three patients (11.5%) in the older group and 18 (8.5%) in the younger group developed complicated diverticulitis (*P* = 0.611). All cases with complicated diverticulitis were grade 2, complicated diverticulitis with small abscesses. There was no patient with diverticulitis more severe than grade 2. All patients were discharged without death or long‐term sequelae. Immunosuppressants were prescribed to five patients as follows: adalimumab to a patient with psoriasis, dexamethasone (0.5 mg/day) to a patient with 21‐hydroxylase deficiency, methotrexate to a patient with rheumatoid arthritis, prednisolone (5 mg/day) to a patient with nephrotic syndrome, and salazosulfapyridine to a patient with rheumatoid arthritis. No patients used non‐steroidal anti‐inflammatory drugs. There was no patient with bacteremia; blood culture was submitted in seven (26.9%) and 45 (21.3%) patients in the older and younger groups, respectively (*P* = 0.515).

### *Univariate comparison*

The results of the univariate comparison are listed in Table 1. Leukocyte count was significantly lower in the older compared with the younger group; leukocyte counts were 10 850/mm^3^ (IQR, 9400–12 000/mm^3^) and 12 600/mm^3^ (IQR, 10 500–15 000/mm^3^) in the older and younger groups, respectively (*P* = 0.0044). The proportion of leukocytosis (defined as >11 000/mm^3^) was also significantly lower in older compared with younger patients (46.2% vs. 68.3%, respectively, *P* = 0.025). The proportion of female patients, left‐sided colonic diverticulitis and immunocompromised patients was significantly higher in the older compared with the younger group (Table 1). Serum CRP level was not significantly different between groups (*P* = 0.718).

**Table 1:** Characteristics of participants

Parameters	Older^†^ (*N* = 26)	Younger^‡^ (*N* = 211)	*P*‐value
Age (years old)	75.5 (67.0–80.0)^¶^	41.0 (32.0–50.0)^¶^	N/A
Female	15 (57.7%)	66 (31.3%)	0.007^*^
Left‐sided colon	15 (57.7%)	42 (19.9%)	<0.001^*^
Previous diverticulitis	8 (30.8%)	43 (20.4%)	0.224
Immunocompromised status	6 (23.1%)	14 (6.6%)	0.004^*^
Diabetes mellitus	1 (3.9%)	6 (2.8%)	0.776
Cancer	5 (19.2%)	2 (1%)	<0.001^*^
Hemodialysis	0	1 (0.5%)	0.725
Immunosuppressant use	0	5 (2.4%)^††^	0.428
Liver cirrhosis	0	1 (0.5%)	0.725
Fever	3 (11.5%)	41 (19.4%)	0.329
Tachycardia	1 (3.9%)	18 (8.6%)	0.407
Shock	0	4 (1.9%)	0.479
Leukocyte count (/mm^3^)	10 850 (9400–12 000) ^¶^	12 600 (10500–15 000) ^¶^	0.004^*^
Leukocytosis	12 (46.2%)	144 (68.3%)	0.025^*^
NLR^§^	6.1 (4.5–11.3)^¶^	6.2 (4.0–8.6)^¶^	0.698
Serum CRP (mg/dL)	5.1 (2.8–10.4)^¶^	5.7 (2.9–10.2)^¶^	0.718
Complicated diverticulitis	3 (11.5%)^‡‡^	18 (8.5%)^‡‡^	0.611
Operated upon	1 (3.9%)	6 (2.8%)	0.776

^†^ Older patients ≥65 years old.

^‡^ Younger patients 16–64 years old.

^§^ NLR was recorded only in 13 older patients and 127 younger patients.

^¶^ Interquartile ranges.

^††^ Prescriptions of immunosuppressants: adalimumab, one patient with psoriasis; dexamethasone (0.5 mg/day), one patient with 21‐hydroxylase deficiency; methotrexate, one patient with rheumatoid arthritis; prednisolone (5 mg/day), one patient with nephrotic syndrome; and salazosulfapyridine, one patient with rheumatoid arthritis.

^‡‡^ All patients had complicated diverticulitis with small abscesses (<6 cm).

* *P* < 0.05.

CRP, C‐reactive protein; N/A, not applicable; NLR, neutrophil–lymphocyte ratio.

### *Logistic regression analysis*

**Figure 1:**
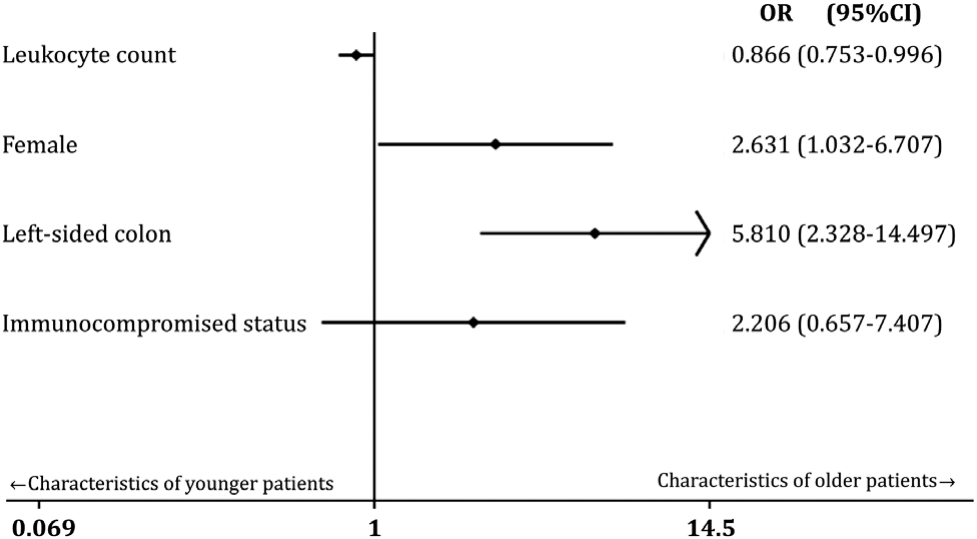
Characteristics of older patients (Forest plot). Leukocyte count has a significantly lower OR. Female and left‐sided colon have significantly higher ORs. Immunocompromised status does not have a significantly high OR. 95% CI, 95% confidence interval; OR, odds ratio.

To adjust for confounding, we selected the leukocyte count, female sex, left‐sided colonic diverticulitis and immunosuppressant use as explanatory variables and performed a logistic regression analysis based on the results of the univariate comparisons. The unadjusted odds ratios and adjusted odds ratios calculated by logistic regression analyses are listed in Table 2. The regression model showed that leukocyte count had an odds ratio that was significantly <1.0, which means that a lower leukocyte count was an independent characteristic of ACD in older patients (Table 2 and Fig. 1). On the other hand, female sex and left‐sided colon diverticulitis had odds ratios of >1.0, which means that female sex and left‐sided colon diverticulitis were independent characteristics of ACD in older patients. Immunocompromised status was not a significant factor (Table 2 and Fig. 1). Receiver operating characteristic analysis revealed that the area under the curve value of the regression model was 0.77, which generally means that the model is moderately accurate.

**Table 2:** Characteristics of older patients (logistic regression analysis)

Variables	Unadjusted OR (95% CI)	Adjusted OR (95% CI)^†^
Leukocyte count	0.832 (0.729–0.949)	0.866 (0.753–0.996)
Female	2.996 (1.305–6.875)	2.631 (1.032–6.707)
Left‐sided colon	5.487 (2.349–12.815)	5.81 (2.328–14.497)
Immunocompromised status	4.221 (1.461–12.200)	2.206 (0.657–7.407)

^†^ Adjusted ORs were calculated by a logistic regression, including leukocyte count, female, left‐sided colon diverticulitis and immunocompromised status as explanatory variables.

CI, confidence interval; OR, odds ratio.

The regression showed good calibration; the chi‐squared result, calculated using the Hosmer–Lemeshow goodness‐of‐fit test, was 6.78 (*P* = 0.560), which means that the model had good fitness. There was no multicollinearity because the VIF for all explanatory variables was ≤1.09 and the mean VIF was 1.06. Optimism, calculated using the bootstrap method, was 0.0001.

Although the leukocyte count was significantly lower in the older group in the regression model, the logistic regression analysis using leukocytosis (categorical variable defined as leukocyte count >11 000/mm^3^) instead of the leukocyte count (continuous variable) showed that leukocytosis was not a significant factor (odds ratio, 0.525; 95% confidence interval, 0.211–1.306; *P* = 0.166).

## Discussion

Our study showed that in patients with ACD, the leukocyte count was significantly lower in the older compared with the younger patients. The result remained significant even after adjusting for confounding variables in a logistic regression model. The study also showed that the prevalence of female patients and left‐sided colonic diverticulitis were significantly higher in older compared with younger patients.

As noted earlier, reactive leukocytosis can be impaired by aging. Indeed, many basic studies have elucidated various mechanisms of neutrophilic immunosenescence such as shortening of half‐life time,^3^, ^4^ decreased T‐cell receptor ligand repertoire diversity,^5^ impaired migration^6^ and neutrophil phagocytic functions.^1^, ^2^ However, the results of previous clinical studies have not necessarily been consistent. Taniguchi *et al*. reported that leukocyte counts in the patients with bacteremia were lower in older patients compared with younger patients.^9^ In contrast, another study reported that leukocyte counts in patients with acute bacterial infections were higher in older patients compared with younger patients.^10^ In hospitalized patients with sepsis, there was no significant difference in the leukocyte counts between older and younger patients.^8^ The different definitions of older patients of the studies may explain the inconsistency: cut‐off ages in previous studies were 65,^8^ 75^10^ and 80 years old.^9^ The definition of leukocytosis was also different, as Yahav *et al*. defined leukocytosis as a leukocyte count >12 000/mm^3^.^10^ The heterogeneity of previous studies due to the broad range of bacterial infection is also an important factor involved in this inconsistency. Previous studies evaluated clinically diagnosed sepsis^8^ or bacterial infection^9^, ^10^ due to any causes instead of specific diagnosis. Considering that our study exclusively evaluated the patients with acute diverticulitis, showed the age‐related difference, and dealt with an accumulation of data from studies on specific infectious diseases, instead of bacterial infection due to various causes, this may contribute to the establishment of the age‐related difference of leukocytosis in response to bacterial infection.

Although logistic regression showed that the absolute leukocyte count was significantly lower in older compared with younger patients, we did not find a significant difference in the proportion (prevalence) of leukocytosis between the groups as an independent factor in multivariate analysis. The prevalence of leukocytosis was significantly lower in older patients in the univariate comparison (*P* = 0.025), which suggests that the absolute leukocyte count should be evaluated instead of leukocytosis because leukocytosis does not have sufficient sensitivity. We think the small sample size in the present study and the small difference (effect size) in the leukocyte counts between the two groups may explain the discrepancy. Further study with a larger sample size may clarify the clinical significance of age‐related differences in leukocytosis.

As another frequently used biomarker for bacterial infection,^12^ we also evaluated the age‐related difference of serum CRP level. However, there was no significant difference between the older and younger groups.

Our study also showed a significantly higher prevalence of left‐sided colonic diverticulitis in older compared with younger patients. This finding is consistent with previous known epidemiology; the Japanese guidelines for colonic diverticular diseases summarized that the incidence of colonic diverticula on the left‐sided colon increases with age to the point where 60% of patients aged ≥70 years have diverticula on the left‐sided colon while 75% of Japanese patients with colonic diverticula who are <50 years of age have diverticula on the right‐sided colon.^19^ Although the mechanism of the age‐related difference of the distribution of the diverticulitis is unknown, we believe that the age‐related increase of left‐sided diverticulitis may reflect the increase of left‐sided diverticula caused by multiple age‐related factors, including effects from lifestyle^20^ and decreases of congenital factors because right‐sided diverticula in Japanese patients have been considered associated with a congenital morphologic predisposition.^21^

There was a significantly high prevalence of female patients in the older patient group in the present study, which is also consistent with a previous study.^22^, ^23^ Although there are few Japanese data on age‐related differences in the sex ratio, epidemiologic studies in other countries revealed that ACD is found predominantly in female patients in the older patient group, while it is found predominantly in male patients in the younger group.^22^, ^23^ For example, ACD showed a remarkable predominance in female patients who were ≥70 years old, while it was predominant in male patients who were <50 years old.^24^ Although no etiology was proposed for the predominance in female older patients, we think that delayed onset and progression of diverticulosis in females compared with males may explain the higher prevalence of females with diverticulitis, a complication of diverticulosis, in older age because the prevalence of diverticular disorders in all age groups is higher in male patients. Male patients may develop diverticular diseases younger than female patients due to a potentially earlier onset of diverticulosis.^22^, ^24^ Because of the lack of Japanese data on age‐related differences in the sex ratio, we think that our study provides new data on the epidemiology of diverticulitis in Japan.

Our study contains a few limitations. Because the present study was performed as a retrospective study that utilized previously collected data, we did not make a preparatory sample size calculation. Therefore, the small sample size may decrease the statistical reliability of the results. For example, we could obtain only 26 older patients and a neutrophil‐to‐lymphocyte ratio from only 13 of 26 (50.0%) and 127 of 221 (57.5%) patients in the older and younger groups, respectively. This small sample size may decrease the statistical reliability of the results. Furthermore, because most participants in the present study had mild diverticulitis, this biased study population might lead to milder cases of leukocytosis. Not only immunocompromised status, but also various underlying disorders have been proposed as a predictive factor of severity.^17^ However, we evaluated only immunocompromised status, including glucocorticoid in this study. We note that our study exclusively evaluated patients with ACD. Because recent guidelines do not recommend the routine use of antibiotics for immunocompetent patients with uncomplicated ACD,^25^ ACD may not be an appropriate example of bacterial infection (i.e., a non‐infectious inflammatory process may play a significant role). However, considering that previous studies evaluating bacterial infection due to various causes showed inconsistent results, we believe that accumulating data from studies focused on specific diseases, as we did in the present study, will contribute to the evaluation of age‐related differences of leukocytosis in response to bacterial infection or acute inflammation. Further study on acute bacterial infection due to other specific causes is certainly warranted.

In conclusion, our logistic regression analysis showed that the leukocyte count was significantly lower in older compared with younger patients with ACD. This result suggests that reactive leukocytosis is impaired and weaker in older compared with younger patients as an expression of immunosenescence. The importance of our study is underscored by the sparsity of clinical data on age‐related differences of reactive leukocytosis in acute infectious/inflammatory disorders. Given the limitations of our study and the discrepancy between basic and clinical study results, further clinical studies on age‐related differences of leukocytosis and neutrophilic immunosenescence in various acute conditions are required.

## Disclosure statement

The authors declare no conflict of interest.
